# An Educational and Physical Program to Reduce Headache, Neck/Shoulder Pain in a Working Community: A Cluster-Randomized Controlled Trial

**DOI:** 10.1371/journal.pone.0029637

**Published:** 2012-01-09

**Authors:** Franco Mongini, Andrea Evangelista, Chantal Milani, Luca Ferrero, Giovannino Ciccone, Alessandro Ugolini, Alessandro Piedimonte, Monica Sigaudo, Elisa Carlino, Emanuela Banzatti, Claudia Galassi

**Affiliations:** 1 Section Headache-Facial Pain, Department of Clinical Pathophysiology, University of Turin, Turin, Italy; 2 Unit of Cancer Epidemiology, San Giovanni Battista Hospital, Turin, Italy; Finnish Institute of Occupational Health, Finland

## Abstract

**Background:**

Noninvasive physical management is often prescribed for headache and neck pain. Systematic reviews, however, indicate that the evidence of its efficacy is limited. Our aim was to evaluate the effectiveness of a workplace educational and physical program in reducing headache and neck/shoulder pain.

**Methodology/Principal Findings:**

Cluster-randomized controlled trial. All municipal workers of the City of Turin, Italy, were invited to participate. Those who agreed were randomly assigned, according to their departments, to the intervention group (IG) or to the control group and were given diaries for the daily recording of pain episodes for 1 month (baseline). Subsequently, only the IG (119 departments, 923 workers) began the physical and educational program, whereas the control group (117 departments, 990 workers) did not receive any intervention. All participants were again given diaries for the daily recording of pain episodes after 6 months of intervention. The primary outcome was the change in the frequency of headache (expressed as the proportion of subjects with a ≥50% reduction of frequency; responder rate); among the secondary outcomes there were the absolute reduction of the number of days per month with headache and neck/shoulder pain. Differences between the two groups were evaluated using mixed-effect regression models. The IG showed a higher responder rate [risk ratio, 95% confidence interval (CI)] for headache (1.58; 1.28 to 1.92) and for neck/shoulder pain (1.53; 1.27 to 1.82), and a larger reduction of the days per month (95% CI) with headache (−1.72; −2.40 to −1.04) and with neck/shoulder pain (−2.51; −3.56 to −1.47).

**Conclusions:**

The program effectively reduced headache and neck/shoulder pain in a large working community and appears to be easily transferable to primary-care settings. Further trials are needed to investigate the program effectiveness in a clinical setting, for highly selected patients suffering from specific headache types.

**Trial Registration:**

ClinicalTrials.gov NCT00551980

## Introduction

Headache disorders are very common, often disabling, costly and associated with overuse of analgesics [1], [2], [3], [4]. Neck pain and its associated disorders also cause a significant health burden in the general population and are experienced by people of all ages [5], [6], [7]. Noninvasive physical management is often prescribed for headache and neck pain. Systematic reviews, however, indicate that the evidence of its efficacy is limited, as most studies have small sample sizes and short follow-ups [8], [9], 10,11,12. A further problem is that attention is usually concentrated on either headache or neck pain, overlooking the fact that they are frequently associated [13], [14].

For some time, we have applied a simple educational and physical program to decrease muscle tension in the head and neck/shoulder area. In our clinical experience, this approach has reduced the frequency and intensity of headache and neck/shoulder pain and the amount of drug intake in a considerable number of patients. In a controlled, non-randomized trial to evaluate the effectiveness of this program administered to a large cohort of public servants, we found that 6 months following treatment, the monthly frequency of headache and neck/shoulder pain was reduced by about 40% in the intervention group (IG) compared to controls [15]. These results were stable at a 12-month follow-up [16].

We have now performed a cluster-randomized controlled trial to evaluate the effectiveness of our program in a much larger cohort of public servants after 6 and 12 months of follow-up. As our program included exercises and visual feedback measures in the workplace, individual randomization was not feasible due to the high level of interference and contamination between subjects working in the same environment. In this paper, we report the results in terms of the primary objective of the study, i.e., to evaluate the effectiveness of the program in reducing the frequency of head and neck/shoulder pain after 6 months.

## Methods

The protocol for this cluster-randomized trial and supporting CONSORT checklist are available as supporting information; see Checklist S1 and Protocol S1.

### Ethics

All eligible subjects were asked to provide their written informed consent. The protocol was approved by the Institutional Review Board of the San Giovanni Battista Hospital of the City of Turin. The study was conducted in accordance with the Declaration of Helsinki.

### Participants

Eligible participants were City of Turin municipal workers (11,780 workers in 444 departments) in May 2007. Workers were contacted by a letter enclosed with the May 2007 pay slip bulletin informing them of the study objectives and requesting their participation. Departments where at least one worker provided informed consent by the end of September 2007 were included as units in the randomization procedure. No exclusion criteria were applied.

### Data collection

In October 2007, all participants were given a one-month diary for the daily recording of the presence and severity of their headache and neck/shoulder pain and their intake of analgesics (by type). The diaries (see the form in the appendix S1) were filled out via a web site, either directly or after printing a copy. The staff monitored the compliance of the participants by identifying those who did not begin the recordings via the web. In some departments, where the majority of participants preferred to fill out the diaries on paper, they were given a paper copy, and one participant, usually of higher hierarchical position, reminded and advocated the recording by the staff and collected the diaries in close envelopes at the end of the month. The diaries were then directly collected by the staff members. The following pain severity scores were used: 0, no pain; 1, very mild pain, not perceived if distracted; 2, mild pain, constantly perceived; 3, moderate to severe pain that permits daily activity; 4, severe pain that impedes any activity; 5, excruciating pain. Some general characteristics and detailed data relative to headache and neck/shoulder pain were collected in a standardized fashion by a questionnaire based on the criteria of the International Classification of Headache Disorders (ICHD) [17] and the International Association for the Study of Pain (IASP) [18]. The following diagnoses were made according to the ICHD and the IASP guidelines: Migraine with or without Aura (M), Tension Type Headache (TTH), Myogenous neck/shoulder pain (MP). Two or more diagnoses in the same subject were possible.

Following the recording of the baseline status in October 2007 (month 1), at the beginning of month 2 the workers were informed of their randomization group, and the IG was shown how to apply the physical and educational program. Both groups filled out the diary again at month 7 (i.e., at month 6 of intervention).

### Intervention

Before administering the program, it was clearly explained to the IG that its aim was to reduce muscle contraction, especially in the cranio-facial-cervical area, and to increase perception of contraction when too elevated. The program consisted of brief shoulder and neck exercises, a relaxation exercise, and instructions on how to reduce parafunction and hyperfunction of the craniofacial and neck muscles during the day.

These instructions were as follows. Relaxation exercise (once–twice a day): Sit down in a comfortable armchair in a quiet room. Let your lower jaw drop completely for 10–15 minutes. Apply warm pads to your cheeks and shoulders. Posture exercises: 1) Stand upright with your heels, hips and nape of the neck against a wall. Without moving the rest of your body, bring your shoulders into contact with the wall and release, rhythmically. 2) With your body and head against the wall, make horizontal movements of the head, forwards and backwards. 3) Cup your hands behind your neck. Stretch your head backwards against counterpressure from your hands. Relax after 2–3 seconds. Perform each exercise 8–10 times in a session; perform a session every 2–3 hours. Visual feedback: Place red labels in strategic sites to remind you to avoid excessive contraction of your head and neck muscles.

The program was delivered to workers by F.M. (professor, MD) and his collaborators: C.M. (DDS), L.F. (DDS), A.U. (DDS), A.P. (Psy.D), M.S. (MD), E.C. (Psy.D), E.B. (DDS). The program was explained in each IG department with a practical demonstration and training to groups of no more than 40 workers. In addition to the red labels for placement around the workplace, other labels were provided for use at home. A written form was also provided with illustrations of the exercises and their instructions. Participants randomized to the IG also had access to a web site to watch a demonstration video. The instructions were repeated twice, after 2 and 4 months. To evaluate the program compliance, a question on the frequency of exercises was added to the month 7 diary, using the following categories: 1) exercises performed fewer than once a week, 2) exercises performed once or twice a week 3) exercises performed three or more days a week, 4) exercises performed exactly as indicated). Due to the low frequency of workers reporting the highest level of compliance, the 3^rd^ and 4^th^ levels were aggregated in one category in the stratified analyses.

### Study outcomes

All the study outcomes were analyzed on the whole population for which both, the baseline and end of the follow up diaries were available (909 subjects in the IG, 972 subjects in the control group).

The primary outcome was the between-group difference in the proportion of symptomatic headache subjects (i.e., subjects with 4 or more days per month with headache during the baseline period) who achieved a ≥50% reduction in pain frequency by month 7 (responder rate). Secondary outcomes were:

- the between-group difference in the proportion of subjects with 4 or more days per month with 1) neck/shoulder pain, 2) headache and/or neck/shoulder pain, 3) analgesic drug consumption during the baseline period, who achieved a ≥50% reduction in pain (or drug consumption) frequency by month 7 (responder rate).
- the between-group mean difference of the change from baseline (month 1) in the number of days per month with 1) headache, 2) neck/shoulder pain, 3) headache and/or neck/shoulder pain, 4) analgesic drug consumption, at month 7.
- the between-group differences in headache or neck/shoulder pain index (average intensity×frequency), where average intensity is the sum of the daily pain intensities in the month, divided by the number of days in the same month.

We did not plan to record any adverse events because they were unexpected based on the literature, our previous study [15], and our clinical experience.

### Sample size

In the previous study [15], all 661 eligible workers were directly informed and contacted at their workplace, and 384 agreed to participate (participation rate 58%). In the present study, the eligible workers were informed and contacted using a less direct strategy, i.e., a letter enclosed in the pay slip bulletin. Therefore, we expected a much lower participation rate with respect to our previous work. Out of the 11,780 eligible workers in 444 departments, we expected a participation rate of about 20%, with a mean of five workers per department; we also assumed a prevalence of symptomatic headache subjects (≥4 days with pain during the baseline) of 50% (in the previous study it was 49% [15]). To detect a difference of 10% (alpha = 0.05, two-tailed) in the proportion of symptomatic subjects who would have a significant reduction (≥50%) of pain frequency (assuming a reduction in 20% of subjects in the IG and a reduction in 10% of subjects in the control group), the expected 2356 subjects (half per group) provided a power of at least 95%, assuming an intracluster correlation (ICC) *ρ* = 0.15 [19].

An accurate estimation of ICC at the time of the study design was unavailable. A previous study [20] reported ICCs calculated from datasets of primary- and secondary-care implementation studies, showing that ICCs of process variables in primary care ranged from 0.05 to 0.15, whereas ICCs for patient outcomes are generally lower than 0.05. Moreover, another work [21] reported the ICCs of 1039 variables evaluated in 31 cluster-based studies, showing that 95% of ICCs were lower than 0.095. Thus, to be more conservative, we estimated the power of the study assuming an ICC of 0.15.

### Sequence generation

The randomization sequence was generated by A.E. (biostatistician) and C.G. (MD), using the SAS Procedure SURVEYSELECT (SAS Institute Inc., Cary, NC, USA, V.8.2). According to the cluster-randomized design, with departments as randomization units, the 271 departments where 2895 workers provided informed consent were stratified for the number of participants and professional groups (Administrative Departments, Educational Departments, Traffic Police Departments) and randomly allocated to the intervention and control groups. To minimize the risk of bias during the baseline data collection, the workers were informed of their randomization group at the end of the first month of the study (baseline period, October 2007), i.e., after the completion of the baseline diaries.

### Statistical analysis

To account for clustering of workers within the departments, the between-group difference of the proportion of symptomatic subjects (≥4 days/month of pain during the baseline period) with a ≥50% reduction in headache pain days at month 7 was evaluated using a mixed-effect logistic regression model [22], considering the department variable as a random effect. The mixed-effect logistic regression model is
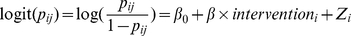
where 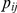 is the probability of the worker *j* in the department *i* achieving a ≥50% reduction in headache pain days and 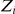 is the random effect of being in the department *i*. 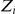 was assumed to be normally distributed, with mean 0 and variance 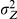.

The odds ratios (ORs) derived from mixed-effect logistic models were converted to risk ratios (RRs) using the method proposed by Zhang and Yu [23]

The ICC is calculated by using this formula:
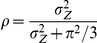
The same method was applied to analyze neck/shoulder pain, headache and/or neck/shoulder pain and analgesic drug consumption. Furthermore, to account for imbalanced factors between groups due to the cluster-based randomization procedure, logistic models were also adjusted for age, gender, education level, job activity, number of workers in each department included in the study, presence of neck/shoulder pain (when the frequency of headache and the use of drug were analyzed) and presence of headache (when the frequency of neck/shoulder pain and the use of drug were analyzed).

Between-group comparisons, based on changes in the frequency of symptoms at the end of the study (month 7 of follow-up) compared with baseline (month 1), were also performed. These differences, calculated for each subject, were compared between the two groups using mixed-effect linear models [22], considering the department as a random effect.

The mixed-effect linear model is
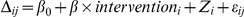
where 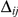 is the difference in the frequency of symptoms between month 7 and baseline for the worker *j* in the department *i*, 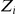 is the random effect of being in the department *i* and 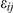 is the random effect at the worker level. 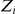 and 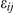 were assumed to be normally distributed, with mean 0 with variance 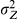 and 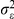, respectively.

The ICC is calculated by using this formula:
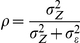
Mixed-effect linear models were also performed adjusting for baseline symptom values of each subject and for the above-mentioned covariates. Exploratory subgroup analyses were performed according to protocol by gender, age, diagnosis, education level and job activity. For each subgroup, adjustments were made for the variables previously listed. Interaction was tested by inserting an interaction term between the IG and the subgroup covariate of interest. The reduction of sample size in subgroup analyses from performing mixed-effect linear models caused some convergence problems in the estimation; therefore, to account for clustering of workers within the departments, subgroup analyses were performed using linear models, adjusting standard errors with the Huber/White/Sandwich estimator [24]. Analyses were performed using STATA version 9.2 (StataCorp LP, College Station, TX), using the commands XTLOGIT for the mixed-effect logistic models, XTMIXED for mixed-effect linear models, and REGRESS with “cluster” option for linear models using the Huber/White/Sandwich estimator.

### Sensitivity analyses

Since 35% of the initially randomized workers did not complete the baseline and end of the follow up diaries, we performed a sensitivity analysis including the whole randomized population (1457 IG, 1438 control group) to assess the impact of this potential bias on the main results.

The crude RRs with corresponding 95% CI for the responder rates were estimated according to the following *scenarios*:

1. *Scenario 1*: the probability of improvement observed in the control group was assigned to the workers not completing the baseline and/or the follow-up diary in both the IG and the control group.
2. *Scenario* 2 (worst *scenario*): the probability of improvement observed in the IG was assigned to the workers not completing the baseline and/or the follow-up diary in the control group, whereas the probability of improvement observed in the control group was assigned to the workers not completing the baseline and/or the follow-up diary in the IG.

## Results

**Figure 1:**
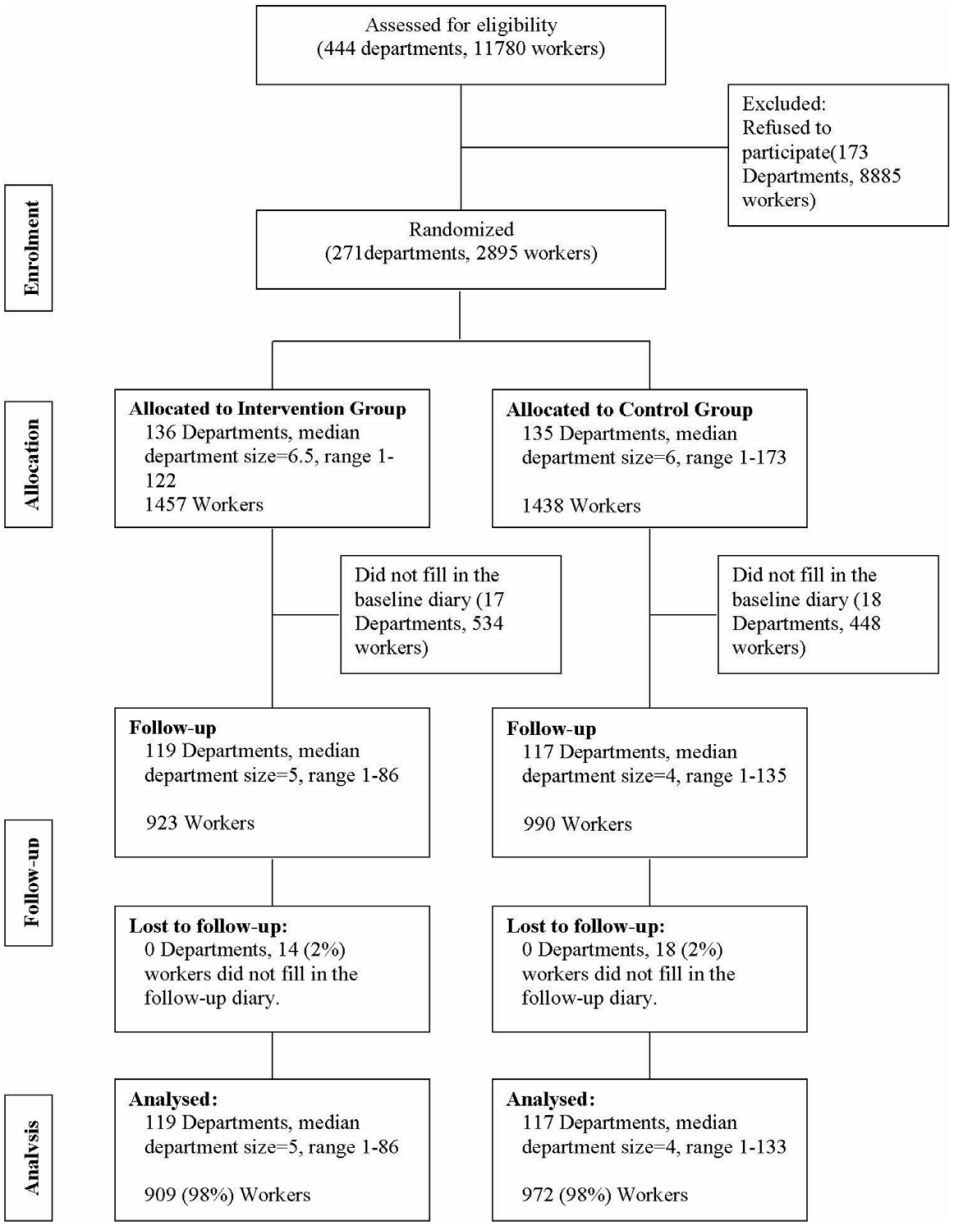
Flow chart.

Figure 1 shows the trial profile. Out of 11,780 eligible workers in 444 departments, 2895 (25%) within 271 departments gave informed consent for participation. However, 534 (37%) workers randomized to IG and 448 (31%) workers randomized to the control group did not complete the baseline diary for month 1. Using information collected in the informed consent form, the group of workers who did not fill out the baseline diary showed a higher prevalence among males (18% vs. 14%) and an higher prevalence among people with a low education level (23% vs. 15%) with respect to workers who filled out the baseline diary. Thus, it was possible to collect the baseline diaries on the remaining 1913 workers within 236 departments (119 departments and 923 workers in the IG, 117 departments and 990 workers in the control group). Overall, the proportion of subjects who filled out the diary directly via the web site was comparable between the two groups (67.7% of IG and 69.8% of controls). At month 7 of the study, six months after randomization, 32 workers (14 IG, 18 control group) did not fill out the follow-up diary. Table 1 summarizes the department and worker characteristics of the analyzed population (909 in the IG, 972 in the control group). The median numbers of workers per department were four and five, respectively, in the intervention and the control group. Although the randomization procedure was cluster-based, the groups were quite similar on demographic and baseline characteristics. The analyzed population was predominantly female, with a median age of 47 years at enrollment. Almost two-thirds of workers were diagnosed as having TTH, and M was diagnosed in 1099 workers (58%); overall, 1471 (78%) workers received a diagnosis of MP.

**Table 1:** Department and worker characteristics of the analyzed population.

	InterventionGroup	ControlGroup	Total
Departments	(N = 119)	(N = 117)	(N = 236)
Size of department, median (range)	5 (1–86)	4 (1–133)	4 (1–133)
Administrative Departments	52 (44%)	52 (44%)	104 (44%)
Traffic Police Departments	15 (13%)	9 (8%)	24 (10%)
Educational Departments	52 (44%)	56 (48%)	108 (46%)

Workers	(N = 909)	(N = 972)	(N = 1881)
Age at the enrollment	48 (43;52)	47 (43;52)	47 (43;52)
Males	112 (12%)	151 (16%)	263 (14%)
Females	797 (88%)	821 (84%)	1618 (86%)
Diagnoses			
M	543 (60%)	556 (57%)	1099 (58%)
TTH	598 (66%)	612 (63%)	1210 (64%)
MP	717 (79%)	754 (78%)	1471 (78%)
Only M	196 (22%)	234 (24%)	430 (23%)
Only TTH	251 (28%)	290 (30%)	541 (29%)
M and TTH	347 (38%)	322 (33%)	669 (36%)
Only Headache	151 (17%)	162 (17%)	313 (17%)
Only MP	74 (8%)	70 (7%)	144 (8%)
Headache and MP	643 (71%)	684 (70%)	1327 (71%)
Job activity			
Office Workers	379 (42%)	401 (41%)	780 (41%)
Educational/Social employees	405 (45%)	392 (40%)	797 (42%)
Traffic Policemen	73 (8%)	80 (8%)	153 (8%)
Other Workers	52 (6%)	99 (10%)	151 (8%)
Education level			
High	134 (15%)	154 (16%)	288 (15%)
Intermediate	639 (70%)	678 (70%)	1317 (70%)
Low	136 (15%)	140 (14%)	276 (15%)
Baseline monthly frequency, mean (SD)			
Headache	7.43 (7.60)	7.28 (7.66)	7.35 (7.63)
Neck/Shoulder pain	11.23 (10.94)	10.80 (10.94)	11.0 (10.94)
Analgesic drug consumption	3.02 (4.89)	2.94 (4.85)	2.98 (4.87)
Subjects with at least 4 days/month with:			
Headache	506 (56%)	529 (54%)	1035 (55%)
Neck/Shoulder pain	550 (61%)	572 (59%)	1122 (60%)
Headache and/or Neck/Shoulder Pain	717 (79%)	741 (76%)	1458 (78%)
Analgesic drug consumption	213 (23%)	214 (22%)	427 (23%)

Data are median (IQR) and n (%) unless otherwise indicated.

TTH = tension-type headache, M = migraine, MP = myogenous neck/shoulder pain.

The headache responder rate was significantly higher in the IG (RR = 1.58, 95% CI 1.28 to 1.92) (Table 2). The ICC for the headache responder rate was 0.039 (95% CI 0.012 to 0.120). Additionally, the probability of achieving a ≥50% decrease of neck/shoulder pain days among symptomatic subjects was greater in the IG (RR = 1.53, 95% CI 1.27 to 1.82). The ICC for the neck/shoulder pain responder rate was 0.029 (95% CI 0.007 to 0.110). When considering the combined outcome (headache and/or neck/shoulder pain) the responder rate was significantly higher in the IG (RR = 1.82, 95% CI 1.52 to 2.15). The ICC for the headache and/or neck/shoulder pain responder rate was 0.044 (95% CI 0.016 to 0.112). An effect was found also in the reduction of drug consumption in the IG (RR = 1.45, 95% CI 1.05 to 1.97). The ICC for the drug consumption responder rate was 0.081 (95% CI 0.030 to 0.199).

**Table 2:** Comparison of the proportion of subjects with (“improved”*) or without (“not improved” ‡) reduction in pain frequency or drug consumption of ≥50% at the end of follow-up (responder rates), between IG and control group.

		Intervention Group	Control Group	RR (95%CI)	RR (95%CI)
		(N = 909)	(N = 972)	Crude	Adjusted§
Headache	Not Improved	672 (74%)	817 (84%)	1	1
	Improved	237 (26%)	155 (16%)	1.58 (1.28, 1.92)	1.58 (1.32, 1.87)
Neck/shoulder pain	Not Improved	636 (70%)	788 (81%)	1	1
	Improved	273 (30%)	184 (19%)	1.53 (1.27, 1.82)	1.53 (1.28, 1.82)
Headache and/or Neck/shoulder pain	Not Improved	590 (65%)	793 (82%)	1	1
	Improved	319 (35%)	179 (18%)	1.82 (1.52, 2.15)	1.83 (1.54, 2.14)
Analgesic Drug consumption	Not Improved	790 (87%)	874 (90%)	1	1
	Improved	119 (13%)	98 (10%)	1.45 (1.05, 1.97)	1.45 (1.03, 1.99)

*“Improved”: subjects with a baseline frequency of ≥4 days/month with pain (or drug consumption) that had a reduction in pain frequency or drug consumption of ≥50% at the end of follow-up.

‡ “Not improved”: includes subjects with ≥4 days/month with pain (or drug consumption) at the baseline with less than 50% of reduction in pain frequency or drug consumption at the end of follow-up, and those subjects that had a baseline frequency of less than 4 days with pain/drug consumption independently from their results.

§ adjusted by age, sex, neck/shoulder pain (when analyzing headache and analgesic drug consumption), headache (when analyzing neck/shoulder pain and analgesic drug consumption), education level, job activity and baseline value of each subject.

A graphic description of the variations in symptom frequencies is reported in the appendix S2. The frequency of the outcomes in the analyzed population, as well as the mean differences for each group and their absolute between-group differences, both crude and adjusted, are summarized in Table 3. For all these end-points associated with headache and neck/shoulder pain, the IG subjects achieved meaningful and statistically significant improvements by the end of the follow-up compared to controls. Mean intervention effects (days per month, 95% CI) when comparing the change from baseline at month 7 were headache frequency −1.72 (−2.40 to −1.04) and frequency of neck/shoulder pain −2.51 (−3.56 to −1.47). Moreover, the headache or neck/shoulder pain index significantly decreased in the IG. For drug consumption, the reduction of frequency in the IG was statistically significant considering the overall group of workers, but not among workers with ≥4 days/month of drug consumption. Similar results were obtained using the Huber/White/Sandwich estimator to account for clustering: headache frequency −1.78 (95% CI, −2.45 to −1.11); frequency of neck/shoulder pain −2.82 (95% CI, −3.82 to −1.82); frequency of headache and/or neck/shoulder pain −3.00 (95% CI, −3.97 to −2.04) frequency of analgesic drugs consumption −0.42 (95% CI, −0.88 to 0.04).

**Table 3:** Effects of the intervention (change from baseline in the number of days with pain or drug consumption at month 7) on the study outcomes.

		Intervention Group (N = 909)		Control Group (N = 972)		Between group differences (Intervention vs Control)	
		Baseline(mean)	Within group difference(mean, 95%CI)	Baseline(mean)	Within group difference(mean, 95%CI)	Crude(mean, 95%CI)	Adjusted*(mean, 95%CI)
Headache	Days with headache, mean	7.42	−2.53 (−3.01, −2.04)	7.28	−0.81 (−1.29, −0.33)	−1.72 (−2.40, −1.04)	−1.63 (−2.20, −1.07)
	among subjects with at least 4 days/month with headache	12.00	−4.70 (−5.44, −3.96)	12.11	−2.47 (−3.21, −1.72)	−2.23 (−3.28, −1.18)	−2.15 (−3.10, −1.21)
	Headache Index (FxI)	0.56	−0.17 (−0.21, −0.14)	0.56	−0.03 (−0.07, 0.00)	−0.14 (−0.19, −0.09)	−0.14 (−0.19, −0.10)
	among subjects with at least 4 days/month with headache	0.90	−0.33 (−0.40, −0.27)	0.92	−0.15 (−0.21, −0.08)	−0.19 (−0.28, −0.09)	−0.19 (−0.27, −0.10)
Neck/shoulder pain	Days with neck/shoulder pain, mean	11.23	−3.23 (−3.97, −2.50)	10.80	−0.72 (−1.46, 0.02)	−2.51 (−3.56, −1.47)	−2.40 (−3.34, −1.47)
	among subjects with at least 4 days/month with neck/shoulder pain	17.78	−6.15 (−7.26, −5.04)	17.63	−2.88 (−4.00, −1.76)	−3.27 (−4.85, −1.70)	−3.30 (−4.70, −1.89)
	Neck/shoulder pain Index (FxI)	0.80	−0.27 (−0.33, −0.22)	0.75	−0.02 (−0.08, 0.03)	−0.25 (−0.33, −0.17)	−0.23 (−0.31, −0.15)
	among subjects with at least 4 days/month with neck/shoulder pain	1.27	−0.21 (−0.27, −0.16)	1.23	−0.06 (−0.12, −0.01)	−0.36 (−0.48, −0.23)	−0.34 (−0.45, −0.22)
Headache and/orNeck/shoulder pain	Days with headache and/or neck/shoulder pain, mean	13.84	−3.93 (−4.64, −3.21)	13.61	−1.28 (−2.00, −0.56)	−2.65 (−3.66, −1.63)	−2.57 (−3.52, −1.63)
	among subjects with at least 4 days/month with headache and/or neck/shoulder pain	17.04	−5.41 (−6.26, −4.57)	17.30	−2.33 (−3.19, −1.47)	−3.08 (−4.29, −1.87)	−3.29 (−4.39, −2.18)
Analgesic Drug consumption	Days with analgesic drug consumption, mean	3.02	−0.87 (−1.19, −0.55)	2.94	−0.38 (−0.70, −0.06)	−0.49 (−0.94, −0.03)	−0.43 (−0.77, −0.08)
	among subjects with at least 4 days/month with analgesic drug consumption	9.55	−4.70 (−5.63, −3.76)	9.78	−3.98 (−4.93, −3.03)	−0.72 (−2.05, 0.62)	−0.83 (−1.95, 0.28)

*Adjusted by age, sex, neck/shoulder pain (when analyzing headache and analgesic drug consumption), headache (when analyzing neck/shoulder pain and analgesic drug consumption), education level, and job activity.

**Figure 2:**
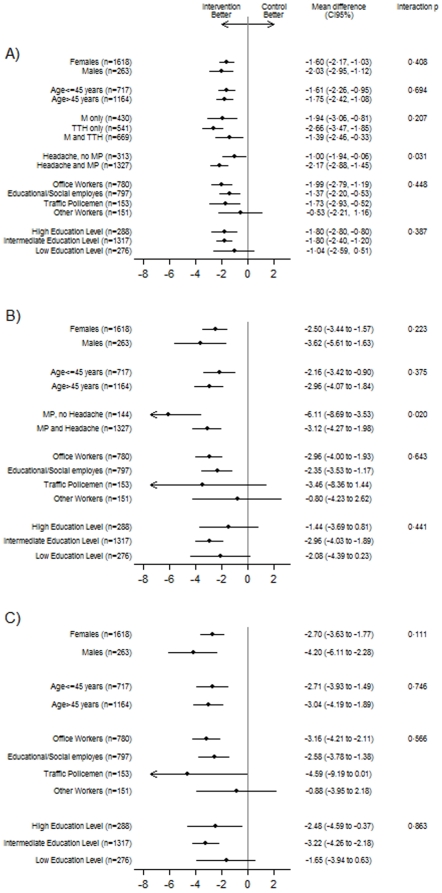
Mean differences (days/month) between groups in the changes from baseline (month 7 vs. month 1) of the frequency of headache (panel A), neck/shoulder pain (panel B), headache and/or neck/shoulder pain (panel C), by subgroups.

Figure 2 reports the effects of treatment on the frequency of headache, neck/shoulder pain and the combined outcome (headache and/or neck/shoulder pain) by subgroups. Reduction of headache frequency seemed to be more evident in workers with a diagnosis of M and/or TTH associated with MP. For neck/shoulder pain a higher reduction of frequency was found in workers with a diagnosis of MP not associated with headache.

The self-reported compliance in the IG group was: 23% low (if exercises were performed fewer than once a week), 54% medium (if exercises were performed once or twice a week), 22% high (if they were performed three or more days a week [19%] or exactly as indicated [3%]).

In the IG, the reduction of the monthly frequency of days with pain was greater among workers with a medium or high level of compliance compared with those with a lower compliance. For headache pain, mean change of frequency from baseline was −1.33 (−2.26 to −0.39) for low compliance, −2.59 (−3.21 to −1.98) for medium compliance, and −3.31 (−4.25 to −2.36) for high compliance. For neck/shoulder pain, mean change of frequency from baseline was −0.95 (−2.40 to 0.50) for low compliance, −3.46 (−4.43 to −2.49) for medium compliance, −4.67 (−6.14 to −3.20) for high compliance. Adjusted mean differences when comparing high vs. low compliance were headache frequency −1.68 (−2.77 to −0.59) and frequency of neck/shoulder pain −3.52 (−5.20 to −1.83). Adjusted mean differences when comparing medium vs. low compliance were headache frequency −0.92 (−1.84 to 0.00) and frequency of neck/shoulder pain −2.18 (−3.60 to −0.77).

For all outcomes analyzed, there were no substantial differences between crude and adjusted estimates of intervention effects (Tables 2 and 3).

The sensitivity analyses performed on the whole randomized population demonstrate that the efficacy of the intervention on headache and neck/shoulder pain persists also in the worst *scenario* (Table 4).

**Table 4:** Results (responder rates) of the sensitivity analyses performed on the whole randomized population according to two different *scenarios*.

		*Scenario 1* *			*Scenario 2* **		
		Intervention Group	Control Group	RR (95%CI)	Intervention Group	Control Group	RR (95%CI)
		(N = 1457)	(N = 1438)		(N = 1457)	(N = 1438)	
Headache	Not Improved	1132 (78%)	1208 (84%)	1	1132 (78%)	1162 (81%)	1
	Improved	325 (22%)	230 (16%)	1.39 (1.20, 1.62)	325 (22%)	276 (19%)	1.16 (1.01, 1.34)
Neck/shoulder pain	Not Improved	1080 (74%)	1165 (81%)	1	1080 (74%)	1114 (77%)	1
	Improved	377 (26%)	273 (19%)	1.36 (1.19, 1.56)	377 (26%)	324 (23%)	1.15 (1.01, 1.31)
Headache and/orNeck/shoulder pain	Not Improved	1039 (71%)	1175 (82%)	1	1039 (71%)	1096 (76%)	1
	Improved	418 (29%)	263 (18%)	1.57 (1.37, 1.80)	418 (29%)	342 (24%)	1.21 (1.07, 1.36)
Analgesic Drug consumption	Not Improved	1283 (88%)	1293 (90%)	1	1283 (88%)	1279 (89%)	1
	Improved	174 (12%)	145 (10%)	1.18 (0.96, 1.46)	174 (12%)	159 (11%)	1.08 (0.88, 1.32)

*Scenario 1: the probability of improvement observed in the control group was assigned to the workers not completing the baseline and/or the follow-up diary in both the IG and the control group.

**Scenario 2 (*worst scenario*): the probability of improvement observed in the IG was assigned to the workers not completing the baseline and/or the follow-up diary in the control group, whereas the probability of improvement observed in the control group was assigned to the workers not completing the baseline and/or the follow-up diary in the IG.

## Discussion

In this cluster-randomized controlled trial, we have demonstrated the effectiveness of our educational and physical program in reducing headache and neck/shoulder pain in a large working community. Among these workers, the proportion of those with at least 4 days/month with pain achieving a ≥50% reduction of days with headache (main outcome of the study) or neck/shoulder pain was increased by at least 1.5 times in the IG compared to controls. Overall, the benefit of the intervention was consistent for all examined end-points associated with headache and neck/shoulder pain, although it was slightly less pronounced for the reduction of drug consumption. An absolute mean reduction of about 2 days/month of pain was observed in the IG subjects compared to controls. The benefit of the intervention was significantly related to the level of compliance.

Previous studies have assessed the efficacy of noninvasive physical management in reducing the frequencies of different types of headache and neck pain, with conflicting results [8], [9], [10], [11], [12], [25], [26], [27], [28], [29], [30]. In a Cochrane systematic review of trials aimed at quantifying the effects of non-invasive physical treatments for chronic/recurrent headaches, Bronfort et al. [9] found 10 good-quality trials, but they could not pool the results because of the high heterogeneity between the studies. The number of patients included in the trials ranged from 12 to 218, and in only three trials was the follow-up longer than 3 months. In a recent Cochrane systematic review to assess whether patient education strategies benefit adults with neck pain, Haines et al. examined 10 selected trials and concluded that no effectiveness for educational interventions for neck pain had been shown [30]. The authors emphasized that in future research, further attention to methodological quality is necessary.

Our study has several strengths. We used a pragmatic approach, with a much larger sample size and a longer follow-up than in most previous studies, and assessed clinically relevant outcomes [9], [30], [31]. Moreover, in our study, head and neck/shoulder pain were investigated jointly. A further strength of the study is the assessment of an association between the level of compliance and the magnitude of the intervention effect.

In this trial the outcomes were not evaluable for about one-third of the randomized workers who did not fill out the baseline (and end of the follow up) diary, even though they initially gave consent to participation. This major limitation was addressed with sensitivity analyses, applying two hypothetical *scenarios*. Even in the worst (and very unlikely) *scenario*, a significant, albeit reduced, benefit of the intervention on headache and neck/shoulder pain was evident; this indicates that possible attrition bias, if present, cannot explain the observed results. Another weakness of our study is that neither the subjects nor the researchers were blinded to the group allocation. However, in physical and behavioral exercise studies, double-blinding is impossible for most interventions, and effective single-blinding is also difficult to achieve in most cases [31].

The prevalence of subjects suffering from frequent pain episodes at the baseline suggests a selective participation in the trial, since subjects with more frequent episodes might have been more interested in participating than those with no or occasional pain.

Although we are not able to definitively confirm that information was recorded on a daily basis by all subjects, we think that a retrospective compilation of the diaries was unlikely because of the monitoring of the compliance by the staff and some participants. Furthermore, the method of filling out the diaries (web or paper) did not differ between intervention and control groups. Finally, even in case of a possible retrospective compilation of the diaries by a subgroup of subjects, available evidence from previous validation studies confirm a high degree of accuracy of the patient recall of headache frequency [32], [33], [34].

As previously observed [15], the improvement in the pain frequency in the control group during the study period, i.e., from October to April, could have been related to seasonal variations and to the subjects' expectations, which were reinforced by keeping a diary. Indeed, the workers of the control group were informed that they would start the program subsequently (at month 8).

Some explanations of the positive effects of the program may be suggested. In our study, management consisted of a simple, self-administered educational and exercise program performed alone at the workplace and at home. Indeed, the daily time required to perform the program is limited, and easily compatible with most occupations. Although simple, our program, in contrast to the majority of those applied in other studies, may have a positive interaction between educational and exercise components. The educational aspects consist in a clear discussion of the major aspects of the problem; in periodical instruction reinforcement, including reinforcement by the more motivated subjects of the working community; and in the application of visual feedback at the workplace and at home. These aspects probably explain also the lower rate of drop-outs compared to other trials. We found that medium-high compliance was accompanied by a greater effect; however, some efficacy was observed also in those whose compliance was low. This could be explained by the fact that the educational aspects of the program were the same for all the participants. Most subjects with a low level of compliance may have acquired the ability to maintain their craniocervical muscles at a lower contraction level during the day. Beneficial effects may have resulted partly from mechanisms involving expectation and conscious anticipation [35].

The results obtained in this study may be of high clinical relevance. Our study shows that a low-cost, low-intensity educational and physical program is effective in reducing head and neck/shoulder pain and possibly analgesic drug consumption in large working populations. This approach seems easily transferable to primary care settings. Further trials are needed to investigate if this program is also effective in a clinical setting, for highly selected patients suffering from specific headache types.

## Supporting Information

Checklist S1 CONSORT Checklist.
(DOC)

Protocol S1 Trial Protocol.
(PDF)

Appendix S1 Diary form.
(PDF)

Appendix S2 Distribution of baseline and follow-up frequency of the number of days with pain and drug consumption.
(DOC)

## References

[1] Stovner LJ, Zwart JA, Hagen K, Terwindt GM, Pascual J (2006). Epidemiology of headache in Europe.. Eur J Neurol.

[2] Wiendels NJ, Knuistingh Neven A, Rosendaal FR, Spinhoven P, Zitman FG (2006). Chronic frequent headache in the general population: prevalence and associated factors.. Cephalalgia.

[3] Stovner L, Hagen K, Jensen R, Katsarava Z, Lipton R (2007). The global burden of headache: a documentation of headache prevalence and disability worldwide.. Cephalalgia.

[4] Steiner TJ (2004). Lifting the burden: The global campaign against headache.. Lancet Neurol.

[5] Ghaffari M, Alipour A, Farshad AA, Yensen I, Vingard E (2006). Incidence and recurrence of disabling low back pain and neck-shoulder pain.. Spine (Phila Pa 1976).

[6] Riddle DL, Schappert SM (2007). Volume and characteristics of inpatient and ambulatory medical care for neck pain in the United States: data from three national surveys.. Spine (Phila Pa 1976).

[7] Hogg-Johnson S, van der Velde G, Carroll LJ, Holm LW, Cassidy JD (2008). The burden and determinants of neck pain in the general population: results of the Bone and Joint Decade 2000–2010 Task Force on Neck Pain and Its Associated Disorders.. Spine (Phila Pa 1976).

[8] Gross AR, Aker PD, Goldsmith CH, Peloso P (2000). Patient education for mechanical neck disorders.. Cochrane Database Syst Rev.

[9] Bronfort G, Nilsson N, Haas M, Evans R, Goldsmith CH (2004). Non-invasive physical treatments for chronic/recurrent headache.. Cochrane Database Syst Rev.

[10] Kay TM, Gross A, Goldsmith C, Santaguida PL, Hoving J (2005). Exercises for mechanical neck disorders.. Cochrane Database Syst Rev.

[11] Biondi DM (2005). Physical treatments for headache: a structured review.. Headache.

[12] Hurwitz EL, Carragee EJ, van der Velde G, Carroll LJ, Nordin M (2008). Treatment of neck pain: noninvasive interventions: results of the Bone and Joint Decade 2000–2010 Task Force on Neck Pain and Its Associated Disorders.. Spine (Phila Pa 1976).

[13] Grimmer K, Nyland L, Milanese S (2006). Repeated measures of recent headache, neck and upper back pain in Australian adolescents.. Cephalalgia.

[14] Sjaastad O, Wang H, Bakketeig LS (2006). Neck pain and associated head pain: persistent neck complaint with subsequent, transient, posterior headache.. Acta Neurol Scand.

[15] Mongini F, Ciccone G, Rota E, Ferrero L, Ugolini A (2008). Effectiveness of an educational and physical programme in reducing headache, neck and shoulder pain: a workplace controlled trial.. Cephalalgia.

[16] Mongini F, Evangelista A, Rota E, Ferrero L, Ugolini A (2009). Long-term benefits of an educational and physical program on headache, and neck and shoulder pain, in a working community.. J Pain.

[17] (2004). The International Classification of Headache Disorders: 2nd edition.. Cephalalgia.

[18] (1986). Classification of chronic pain. Descriptions of chronic pain syndromes and definitions of pain terms. Prepared by the International Association for the Study of Pain, Subcommittee on Taxonomy.. Pain.

[19] Donner A, Birkett N, Buck C (1981). Randomization by cluster. Sample size requirements and analysis.. Am J Epidemiol.

[20] Campbell M, Grimshaw J, Steen N (2000). Sample size calculations for cluster randomised trials. Changing Professional Practice in Europe Group (EU BIOMED II Concerted Action).. Journal of health services research & policy.

[21] Adams G, Gulliford MC, Ukoumunne OC, Eldridge S, Chinn S (2004). Patterns of intra-cluster correlation from primary care research to inform study design and analysis* 1.. Journal of clinical epidemiology.

[22] Snijders TAB Bosker RJ 1999 Multilevel analysis : an introduction to basic and advanced multilevel modeling London SAGE[ix], 266

[23] Zhang J, Yu KF (1998). What's the Relative Risk?. JAMA: the journal of the American Medical Association.

[24] White H (1980). A heteroskedasticity-consistent covariance matrix estimator and a direct test for heteroskedasticity.. Econometrica.

[25] Viljanen M, Malmivaara A, Uitti J, Rinne M, Palmroos P (2003). Effectiveness of dynamic muscle training, relaxation training, or ordinary activity for chronic neck pain: randomised controlled trial.. Bmj.

[26] Ylinen J, Takala EP, Nykanen M, Hakkinen A, Malkia E (2003). Active neck muscle training in the treatment of chronic neck pain in women: a randomized controlled trial.. Jama.

[27] Sjogren T, Nissinen KJ, Jarvenpaa SK, Ojanen MT, Vanharanta H (2005). Effects of a workplace physical exercise intervention on the intensity of headache and neck and shoulder symptoms and upper extremity muscular strength of office workers: a cluster randomized controlled cross-over trial.. Pain.

[28] Chiu TT, Lam TH, Hedley AJ (2005). A randomized controlled trial on the efficacy of exercise for patients with chronic neck pain.. Spine (Phila Pa 1976).

[29] Hoving JL, de Vet HC, Koes BW, Mameren H, Deville WL (2006). Manual therapy, physical therapy, or continued care by the general practitioner for patients with neck pain: long-term results from a pragmatic randomized clinical trial.. Clin J Pain.

[30] Haines T, Gross A, Burnie SJ, Goldsmith CH, Perry L (2009). Patient education for neck pain with or without radiculopathy.. Cochrane Database Syst Rev.

[31] Rains JC, Penzien DB, McCrory DC, Gray RN (2005). Behavioral headache treatment: history, review of the empirical literature, and methodological critique.. Headache.

[32] McKenzie JA, Cutrer FM (2009). How Well Do Headache Patients Remember? A Comparison of Self-Report Measures of Headache Frequency and Severity in Patients with Migraine.. Headache: The Journal of Head and Face Pain.

[33] Niere K, Jerak A (2004). Measurement of headache frequency, intensity and duration: comparison of patient report by questionnaire and headache diary.. Physiotherapy Research International.

[34] Van den Brink M, Bandell-Hoekstra E, Abu-Saad HH (2001). The occurrence of recall bias in pediatric headache: a comparison of questionnaire and diary data.. Headache: The Journal of Head and Face Pain.

[35] Amanzio M, Benedetti F (1999). Neuropharmacological dissection of placebo analgesia: expectation-activated opioid systems versus conditioning-activated specific subsystems.. J Neurosci.

