# Supplementary material for: An Educational and Physical Program to Reduce Headache, Neck/Shoulder Pain in a Working Community: A Cluster-Randomized Controlled Trial
Source: PLoS One. 2012 Jan 9;7(1):e29637. doi: 10.1371/journal.pone.0029637 (PMC3253792; doi:10.1371/journal.pone.0029637)
Supplement: Checklist S1 — CONSORT Checklist. (DOC) [file pone.0029637.s001.doc]

NOTE: From March 2010, a new checklist, deriving from the updated guidelines for reporting parallel group randomised trials, is available. However, the update for the extension to the CONSORT Statement for cluster randomised trials is still unavailable. Thus, we provide a filled checklist based on the available extension for cluster randomised trials, including the three items (23, 24, 25) added to the new CONSORT checklist.

CONSORT Statement,

**Checklist of items to include when reporting a cluster randomised trial**

| ***PAPER SECTION* And topic** | **Item** | **Descriptor** | **Reported in Section** |
| --- | --- | --- | --- |
| *TITLE & ABSTRACT* | 1 | How participants were allocated to interventions (eg random allocation, randomised, or randomly assigned), specifying that allocation was based on clusters. | Title/Abstract |
| *INTRODUCTION* Background | 2 | Scientific background and explanation of rationale, including the rationale for using a cluster design | Introduction |
| *METHODS* Participants | 3 | Eligibility criteria for participants and clusters and the settings and locations where the data were collected | Methods: participants |
| Interventions | 4 | Precise details of the interventions intended for each group, whether they pertain to the individual level, the cluster level, or both, and how and when they were actually administered. | Methods: intervention |
| Objectives | 5 | Specific objectives and hypotheses and whether they pertain to the individual level, the cluster level, or both. | Introduction |
| Outcomes | 6 | Report clearly defined primary and secondary outcome measures, whether they pertain to the individual level, the cluster level, or both, and, when applicable, any methods used to enhance the quality of measurements (eg multiple observations, training of assessors) | Methods: Data collection, Study Outcomes |
| Sample size | 7 | How total sample size was determined (including method of calculation, number of clusters, cluster size, a coefficient of intracluster correlation (ICC or k), and an indication of its uncertainty) and, when applicable, explanation of any interim analyses and stopping rules. | Methods: Sample Size |
| Randomization -- Sequence generation | 8 | Method used to generate the random allocation sequence, including details of any restriction (eg blocking, stratification, matching) Allocation. | Methods: Sequence generation |
| Randomization -- Allocation concealment | 9 | Method used to implement the random allocation sequence, specifying that allocation was based on clusters rather than individuals and clarifying whether the sequence was concealed until interventions were assigned. | Methods: Sequence generation |
| Randomization -- Implementation | 10 | Who generated the allocation sequence, who enrolled participants, and who assigned participants to their groups. | Methods: Sequence generation  Contributors |
| Blinding (masking) | 11 | Whether participants, those administering the interventions, and those assessing the outcomes were blinded to group assignment. If done, how the success of blinding was evaluated. | Discussion |
| Statistical methods | 12 | Statistical methods used to compare groups for primary outcome(s) indicating how clustering was taken into account; methods for additional analyses, such as subgroup analyses and adjusted analyses. | Methods: Statistical analysis |
| *RESULTS*  Participant flow | 13 | Flow of clusters and individual participants through each stage. Specifically, for each group report the numbers of clusters and participants randomly assigned, receiving intended treatment, completing the study protocol, and analysed for the primary outcome. Describe protocol deviations from study as planned, together with reasons. | Results;  Fig. 1 |
| Recruitment | 14 | Dates defining the periods of recruitment and follow up. | Methods: Participants, Data Collection |
| Baseline data | 15 | Baseline information for each group for the individual and cluster levels as applicable. | Results;  Table 1 |
| Numbers analyzed | 16 | Number of clusters and participants (denominator) in each group included in each analysis and whether the analysis was by intention to treat. | Methods: Statistical analysis;  Fig. 1;  Table 1 |
| Outcomes and estimation | 17 | For each primary and secondary outcome, a summary of results for each group for the individual or cluster level as applicable, and the estimated effect size and its precision (eg 95% confidence interval) and a coefficient of intracluster correlation (ICC or k) for each primary outcome. | Results;  Table 2;  Table 3 |
| Ancillary analyses | 18 | Address multiplicity by reporting any other analyses performed, including subgroup analyses and adjusted analyses, indicating those prespecified and those exploratory. | Methods: Statistical analysis; Sensitivity Analysis;  Results; Figure 2: Table 4 |
| Adverse events | 19 | All important adverse events or side effects in each intervention group. | Methods: Study oucomes. |
| *DISCUSSION* Interpretation | 20 | Interpretation of the results, taking into account study hypotheses, sources of potential bias or imprecision and the dangers associated with multiplicity of analyses and outcomes. | Discussion |
| Generalizability | 21 | Generalisability (external validity) to individuals and/or clusters (as relevant) of the trial findings. | Discussion |
| Overall evidence | 22 | General interpretation of the results in the context of current evidence. | Discussion |
| OTHER INFORMATION  Registration | 23 | Registration number and name of trial registry | Abstract |
| Protocol | 24 | Where the full trial protocol can be accessed, if available | Available on request |
| Funding | 25 | Sources of funding and other support (such as supply of drugs), role of funders | Funding |

**www.consort-statement.org**
