# Supplementary material for: An Educational and Physical Program to Reduce Headache, Neck/Shoulder Pain in a Working Community: A Cluster-Randomized Controlled Trial
Source: PLoS One. 2012 Jan 9;7(1):e29637. doi: 10.1371/journal.pone.0029637 (PMC3253792; doi:10.1371/journal.pone.0029637)
Supplement: Protocol S1 — Trial Protocol. (PDF) [file pone.0029637.s002.pdf]

Brief title: A RCT on the effectiveness of a cognitive and physical intervention to reduce head and muscle pain in working communities

Official Title: EFFECTIVENESS OF A COGNITIVE AND PHYSICAL PROGRAMME IN REDUCING HEADACHE, NECK AND SHOULDER PAIN IN AN EXTENSIVE WORKING COMMUNITY. A CLUSTER-RANDOMISED-CONTROLLED TRIAL

Principal Investigator:

Franco Mongini

Study Committee:

Chantal Milani

Luca Ferrero

Alessandro Ugolini

Department of Clinical Pathophysiology, Headache and Facial Pain Unit, University of Turin, Turin, Italy

Study design and statistical analysis:

Giovannino Ciccone

Claudia Galassi

Andrea Evangelista

Manuela Ceccarelli

Unit of Cancer Epidemiology (CPO-Piemonte), ASO S.Giovanni Battista Turin

Introduction

Headache and pain symptoms related to neck and shoulders are a clinical challenge, as well as a major health problem.

Therapeutic exercise proved to be able to decrease the intensity of pain in the neck-shoulder area (Konijemberg et al., 2001; Ylinen et al., 2003), also in the workplace, but in a few studies, with different methodological approaches, which do not allow a proper comparison of the results.

Muscle tenderness, above all of pericranial muscles, whose pathophysiological role has been widely proved in tension-type headache (Langemark and Olesen, 1987; Jensen et al., 1998), has recently come into question also in migraine as a factor affecting the disease natural history, above all in relation to psychiatric comorbidity (Mongini et al., 2004). Therefore, relieving muscle tenderness by physical exercise is expected to ameliorate also the headache. Recently, in a small sample of office workers, a positive effect of a workplace physical exercise intervention has been preliminarly demonstrated on the intensity either of the neck and shoulder symptoms, or of the headache, in a cluster randomized controlled cross-over study (Sjögren et al., 2005).

Subjects suffering from different headache types usually complain of numerous accompanying symptoms, behavioural and somatic, which may be partially related to psychiatric comorbidity (Maizels and Burchette, 2004), in particular to anxiety and mood disorders, which are common in this population. The relationship between headache and depression or anxiety disorders seems to be bi-directional (Merikangas et al., 1993; Breslau et al., 2003; Zwart et al., 2003). From recent evidence, physical exercise seems to yield an antidepressant efficacy (Ernst et al., 2006; Harris et al., 2006; De Moor et al., 2006)

Many studies were performed to assess the efficacy of non invasive physical interventions in the treatment of different types of headache and cervical pain. Dynamic muscle training and relaxation training are often prescribed for chronic neck pain. However the evidence on their effectiveness is still limited and the results of recent studies are conflicting (Ylinen J et al., 2003; Viljanen M et al., 2003; Sjogren T et al., 2005).

Meta-analyses show that the clinical effectiveness of non-invasive physical treatments for headache and neck pain require further research using scientifically rigorous methods (Borghouts JA et al., 1998; Kjellman GV et al., 1999; Gross AR et al., 2000; Gross AR et al., 2000; Bronfort G et al., 2001; Linton SJ et al., 2001).

In this complex interplay among muscle tension, headache, neck-shoulder pain and psychiatric comorbidity, so as to achieve a better understanding of the role of physical exercise in relieving pain symptoms, longitudinal, controlled studies are required, in which a precise diagnostic categorization of the subjects not only for the headache type, but also for the psychiatric comorbidity is mandatory. In a previous non-randomized-controlled study in which the cognitive and physical programme was applied to a consistent number of office workers, a significant reduction of frequency of pain in the head and neck areas and of analgesic drug consumption was obtained. The present study was designed to confirm the data in a much more extensive working population and applying a randomised design.

Primary objective

- To confirm the effectiveness of a workplace cognitive and physical programme (Intervention), in reducing the frequency of pain involving the head and neck area in a large working community using a randomized design.

Secondary objectives

- To confirm the effectiveness of the Intervention, in reducing the intensity of pain involving the head and neck area in a large working community using a randomized design.
- To confirm the reduction in analgesic drugs consumption after the Intervention in a large working community using a randomized design.
- To evaluate the persistence of the effectiveness of the Intervention after 12 months in a large working community using a randomized design.

Study design

This is a controlled, cluster randomised, interventional trial conducted on the local government employees of the Municipality of Turin. The participants will be distributed in two groups according to local departments distribution. The eligible source population consists of 11780 workers in 444 departments. A cluster-randomised design will be employed to assign the eligible population in two groups (study and control group) stratified for professional groups and number of participants.

Primary outcome

The primary outcome measures are:

- the headache frequency
- the neck and shoulder pain frequency.

The efficacy of the Intervention will be measured in two ways:

a) responders rate. End-point: proportion of subjects with more than 4 days with headache (neck and shoulder pain) during the baseline period that will have reduction in pain frequency of more than 50%, after 6 months.

b) reduction in the frequency of pain. End-point: number of days per month with a headache (neck and shoulders pain), after 6 months.

Secondary outcomes

Secondary outcome measures are:

- the headache index (Intensity x Frequency) after 6 and 12 months, where intensity is the sum of intensity of the daily pain in a month divided by number of days with headache and frequency is number of days with headache in a month.

Further secondary outcome measures are:

- the frequency of analgesic drug consumption after 6 and 12 months
- the frequency of headache (neck and shoulder pain) after 12 months

Inclusion and Exclusion criteria

All the subjects employed at the Municipality of Turin at Septenber, 2007 will be recruited.

Before starting the study, subjects will have to give via web informed consent to participate in the study according to the protocol for data collection (web-based).

Because of the pragmatic design, no exclusion criteria are required for this study.

Study duration

Data from workers will be collected for 13 months (from October 1st, 2007 to October 31st, 2008)

Ethical aspects

This study is oriented to a working community of healthy subjects who individually accept to participate and is totally non invasive (participants will only be instructed with explanations, video projections and printed material how to perform some simple neck and shoulder exercises and how to keep their cranio-cervical muscles reasonably relaxed during the day).

Given the very extensive size of the eligible population acceptance after informed consent and data will be collected via web. To this purpose all participants will receive a personal password and the data will be protected taking account of the suggestions of the legal section of the San Giovanni Hospital. In particular the informatic tools are as follows:

WINDOWS SERVER 2003

This operating system specific for servers improves security for Web transactions

- isolating individual Web application,
- preventing applications from disrupting the Web services or other Web applications on the server
- preventing application failures,
- providing health-monitoring capabilities
- including internal firewall

This is also possible through services included as

- IIS 6.0 (**Internet Information Services),** well known group of services now improved.
- CLR (**Common language runtime):** a software engine that verifies that applications can run without errors and checks security permissions to ensure that code only perform appropriate operations.

SQL Server

Database is based on **SQL Server security model** passing through two stages of authentication: one at the SQL Server level and the other at the database level.
Login names are maintained within the master database in order to make possible the backup after adding new logins to SQL Server. SQL Server logins are associated with user accounts and SQL server provides Authentication through both **Windows authentication mode** and a **Mixed mode**.

ASP.NET

It's an application based on security operations that involves 3 fundamental steps:

- **Authentication:** the process of validating the identity to accepting credentials (e.g. username and password). Requests from the same user ideally are not subject to the authentication process until the user logs out of the web application.

- **Authorization:** users authenticated are allowed to access specific resources.

- **Impersonation:** this process enables an application to ensure the identity of the user and enables a server process to run using the security credentials of the client so that the ASP.NET applications are capable to execute the identity of client on whose behalf they are operating.

Schedule

After informed consent, all participants will be asked to submit data relative to the presence and characteristics of headache and neck and shoulder pain (September 2007), and to keep a diary (web-based) for one month (October 2007, baseline period).

The participants will be randomized (cluster-randomization) in two groups (study and control group) according to departements distribution (September-October 2007).

At month 2 (November 2007) an instruction program will be administered to the study group only. The intervention consists of brief shoulder and neck exercises to be performed several times a day, a relaxation exercise and instructions of how to reduce hyperfunction of the craniofacial and cervical muscles during the day (see: Intervention).

The control group, instead, will not receive any cognitive program.

Both groups will again fill in the diary during month 7 (April 2008).

At beginning of month 8 (May 2008, beginning of the second phase of the study), the same instruction program (Intervention) will be administered to the control group.

Both groups will again fill in the diaries during month 13 (October 2008, end of the study).

The study design according to time flow is reported in Fig.1

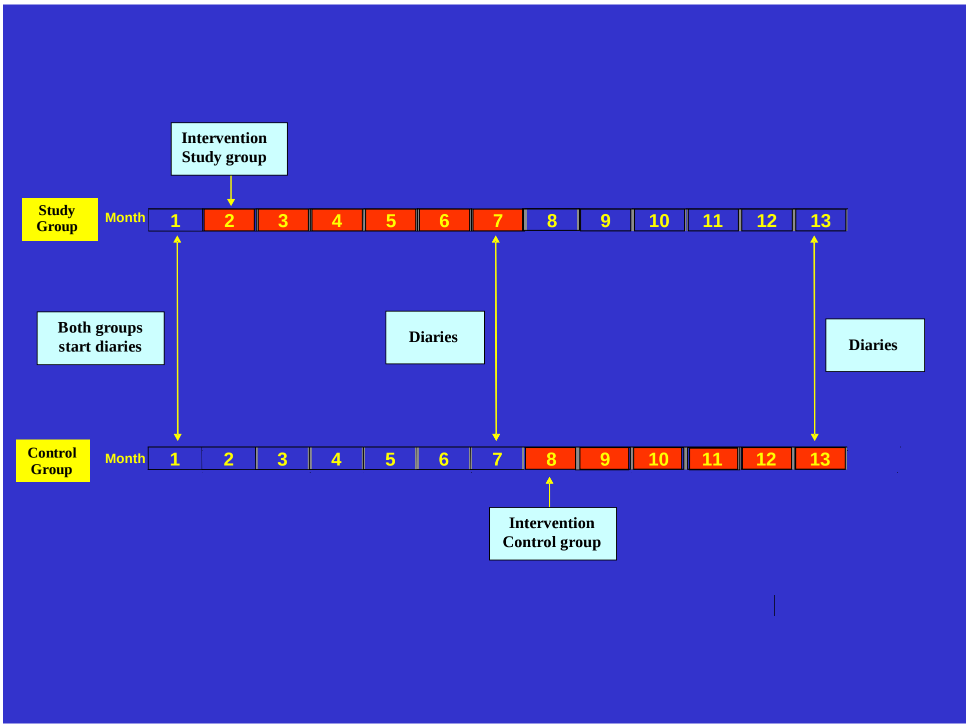

Fig.1 . Study design

- Intervention

The instructions given to the participants of the experimental group (phase 1) and subsequently to the control group (phase 2) will be the following:

a) Relaxation exercise (once or twice a day):
Sit down on a comfortable armchair in a quiet room. Let the mandible drop in a position of maximum relaxation for about 10-15 minutes. Apply warm pads on cheeks and shoulders.

b) Posture exercises : 8-10 times every 2-3 hours
1) Keep an erect position with the tallons, the hips and the nuke against the wall. While the rest of the body does not move bring the shoulders into contact with the wall and release, rhythmically

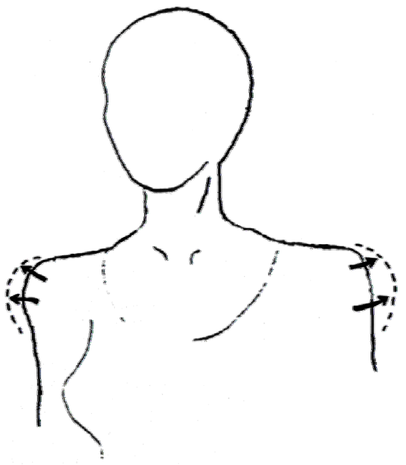

2) While body and head are kept against the wall, make horizontal movements of thehead, forwards and backwards

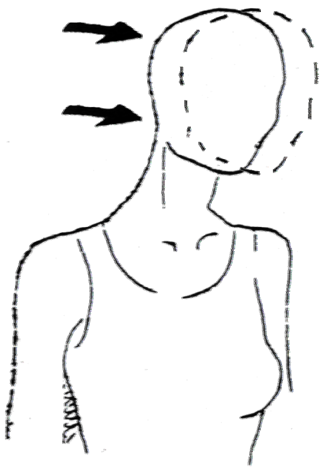

3) After having cupped the hands behind the neck, performs stretching movements of the head backward, with forward counterpressure from the hands. Relaxe after 2-3 seconds.

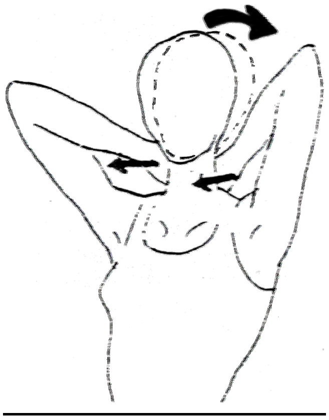

c) Visual feedback:

Place coloured labels in strategic sites at your workplace and at home to remind to keep muscle contraction at a normal level.

The programme will be explained and showed via web and by personal practical demonstrations by a team of trained clinicians three times during months 2-3, 4-5 and 6-7. The subjects in the study group will be asked monthly to rate their compliance level via web.

Controls

In the control group during the first 7 months (phase 1), no Intervention will be performed nor any training instruction provided; the participants will be asked only to keep the diary everyday at months 1 and 7.

At beginning of month 8 (beginning of the second phase), the same Intervention will be administered to the control group, and diaries will be collected again at month 13.

Data collection

All the participants will report in a diary, on a daily basis during months 1,7 and 13, the following information:

- presence or absence of the headache and neck and shoulder pain episodes
- severity (score from 1 to 5) of pain
- drug intake
- for women, menstruation days.

Sample size

Of the eligible 11.780 workers, we will expect a participation rate of 20% of the sample.

The planned 2356 subjects (half per group) will provide a power of 0.95 to detect at least a difference of 10% (alpha= 0.05, two-tails) in the proportion of symptomatic subjects (more than 4 days with pain during the baseline) that will have a significative reduction of pain frequency (assuming a prevalence of symptomatic subjects of 50%, a significative reduction (≥50%) in 20% of subjects in the Intervention group, a significative reduction in 10% of subjects in the Control group, and taking into account the cluster-randomized design of the study, assuming an intracluster correlation $\rho = 0.15$).

Statistical analysis

Subjects who will not give informed consent or will not complete the baseline diary (month 1) will be excluded from the analysis. The data will be analysed according to group distribution.

The **principal analysis** will be conducted between intervention and control group for the first phase of the study (months 1-7; Figure 1, line 1).

Analyses will be performed on subjects for which diaries will be completed for months 1 and 7.

The proportion of subjects with more than 50% reduction in headache days (and in neck and shoulder pain days) at the end of the study period will be assessed and compared between the two groups accounting for the cluster-randomized design of the study.

For each subject, the difference (Delta) in the frequency of symptoms between months 7 and the baseline (month 1) will be calculated; the Deltas between the two groups will be compared with parametric and non-parametric tests, where appropriate, taking into account the cluster-randomized design of the study.

Possible baseline differences between the study group and the controls will be evaluated with parametric and non-parametric tests were appropriate; unbalanced factors will be taken into account in the analysis, if necessary.

Explorative subgroup analyses will be performed by age, gender, severity, compliance, diagnosis, and type of job activity.

A **secondary analysis** (Figure 1, line 2) will be conducted within the intervention group, in order to evaluate the persistence of the efficacy of the intervention; the frequency of pain at months 13 will be compared with that observed at months 7.

A further secondary analysis will be conducted in order to compare the efficacy of the program in the intervention group (end of phase 1) and in control group (end of phase 2).

REFERENCES

American Psychiatric Association. Diagnostic and Statistical Manual of Mental Disorders (DSM-IV) Washington, DC; 1994.

Borghouts JA, Koes BW, Bouter LM. The clinical course and prognostic factors of non-specific neck pain. A systematic review. *Pain* 1998;77: 1-13.

Bronfort G, Evans R, Nelson B, Aker PD, Goldsmith CH, Vernon H. A randomized clinical trial of exercise and spinal manipulation for patients with chronic neck pain. Spine. 2001 Apr 1;26(7):788-97;

De Moor MH, Beem AL, Stubbe JH, Boomsma DI, De Geus EJ. Regular exercise, anxiety, depression and personality: A population-based study. Prev Med. 2006 Jan 23;

Ernst C, Olson AK, Pinel JP, Lam RW, Christie BR. Antidepressant effects of exercise: evidence for an adult-neurogenesis hypothesis? J Psychiatry Neurosci. 2006 Mar;31(2):84-92.

Gross AR, Aker PD, Goldsmith CH, Peloso P. Conservative management of mechanical neck disorders. A systematic overview and meta-analysis. In: *Cochrane Library*, Issue 4. Oxford: Update Software, 2000.

Gross AR, Aker PD, Goldsmith CH, Peloso P. Patient education for mechanical neck disorders. A systematic overview and meta-analysis. In: *Cochrane Library*, Issue 4. Oxford: Update Software, 2000.

Harris AH, Cronkite R, Moos R. Physical activity, exercise coping, and depression in a 10-year cohort study of depressed patients.J Affect Disord. 2006 Mar 16; [Epub ahead of print] PMID: 16545873

International Headache Society, 2004. The International Classification of Headache Disorders. Cephalalgia 2004; 24 (Suppl 1):1-160.

Jensen R, Bendtsen L, Olesen J. Muscular factors are of importance in tension-type headache. Headache 1998;38:10-17

Kjellman GV, Skargren EI, Oberg BE. A critical analysis of randomized clinical trials on neck pain and treatment efficacy. A review of the literature. *Scand J Rehabil Med* 1999;31: 139-52.

Langemark M, Olesen J. Pericranial tenderness in tension headache. A blind, controlled study. Cephalalgia. 1987 Dec;7(4):249-55.

Linton SJ, van Tulder MW. Preventive interventions for back and neck pain problems: what is the evidence? Spine. 2001 Apr 1;26(7):778-87.

Maizels M, Burchette R. Somatic symptoms in headache patients: the influence of headache diagnosis, frequency, and comorbidity. Headache. 2004 Nov-Dec;44(10):983-93.

Mongini F, Ferla E, Gioria A, Simone D, Tarenzi L. Assessment of systemic factors and personality characteristics in tension-type headache and other types of facial pain. In: Olesen J, ed. Tension-type Headache: Classification, Mechanisms and Treatment. New York: Raven Press; 1993;231-36

Mongini F, Ciccone G, Deregibus A, Ferrero L, Mongini T. Muscle tenderness in different headache types and its relation to anxiety and depression. Pain. 2004; 112: 58-63.

Mongini F, Rota E, Deregibus A, Ferrero L, Migliaretti G, Cavallo F, Mongini T, Novello A. Accompanying symptoms and psychiatric comorbidity in migraine and tension-type headache patients". Journal of Psychosomatic Research. 2006; 61 :447-51.

Sjogren T, Nissinen KJ, Jarvenpaa SK, Ojanen MT, Vanharanta H, Malkia EA. Effects of a workplace physical exercise intervention on the intensity of headache and neck and shoulder symptoms and upper extremity muscular strength of office workers: a cluster randomized controlled cross-over trial. Pain. 2005 Jul;116(1-2):119-28.

Ylinen J, Takala EP, Nykanen M, Hakkinen A, Malkia E, Pohjolainen T, Karppi SL, Kautiainen H, Airaksinen O. . Active neck muscle training in the treatment of chronic neck pain in women: a randomized controlled trial. JAMA 2003 May 21;289(19):2509-16.

Viljanen M, Malmivaara A, Uitti J, Rinne M, Palmroos P, Laippala P. Effectiveness of dynamic muscle training, relaxation training, or ordinary activity for chronic neck pain: randomised controlled trial. BMJ 2003 Aug 30;327(7413):475.
