# Supplementary material for: An Educational and Physical Program to Reduce Headache, Neck/Shoulder Pain in a Working Community: A Cluster-Randomized Controlled Trial
Source: PLoS One. 2012 Jan 9;7(1):e29637. doi: 10.1371/journal.pone.0029637 (PMC3253792; doi:10.1371/journal.pone.0029637)
Supplement: Appendix S1 — Diary form. (PDF) [file pone.0029637.s003.pdf]

SEZIONE CEFALEE E DOLORE FACCIALE

CARTELLA SINTOMATOLOGICA QUOTIDIANA

Cognome:

Nome:

Anno | Mese | Giorno

DOLORE ALLA TESTA

	1	2	3	4	5	6	7	8	9	10	11	12	13	14	15	16	17	18	19	20	21	22	23	24	25	26	27	28	29	30	31
5																															
4																															
3																															
2																															
1																															
0																															

DOLORE A COLLO E SPALLE

	1	2	3	4	5	6	7	8	9	10	11	12	13	14	15	16	17	18	19	20	21	22	23	24	25	26	27	28	29	30	31
5																															
4																															
3																															
2																															
1																															
0																															

	1	2	3	4	5	6	7	8	9	10	11	12	13	14	15	16	17	18	19	20	21	22	23	24	25	26	27	28	29	30	31
**MESTRUAZIONI**																															

ASSUNZIONE FARMACI

	1	2	3	4	5	6	7	8	9	10	11	12	13	14	15	16	17	18	19	20	21	22	23	24	25	26	27	28	29	30	31

8 493064 758222
