# Supplementary material for: An Educational and Physical Program to Reduce Headache, Neck/Shoulder Pain in a Working Community: A Cluster-Randomized Controlled Trial
Source: PLoS One. 2012 Jan 9;7(1):e29637. doi: 10.1371/journal.pone.0029637 (PMC3253792; doi:10.1371/journal.pone.0029637)
Supplement: Appendix S2 — Distribution of baseline and follow-up frequency of the number of days with pain and drug consumption. (DOC) [file pone.0029637.s004.doc]

**Figure 1: Distribution of baseline and month 7 frequency of the number of days with headache pain, by group.**

**Figure 2: Distribution of baseline and month 7 frequency of the number of days with neck/shoulder pain, by group.**

**Figure 3: Distribution of baseline and month 7 frequency of the number of days with headache and/or neck/shoulder pain, by group.**

**Figure 4: Distribution of baseline and month 7 frequency of the number of days with analgesic drug consumption, by group.**
